# Supplementary material for: Extracting reproducible components from electroencephalographic responses to transcranial magnetic stimulation with group task-related component analysis
Source: Imaging Neurosci (Camb). 2026 Jan 8;4:IMAG.a.1085. doi: 10.1162/IMAG.a.1085 (PMC12784221; doi:10.1162/IMAG.a.1085)
Supplement: Supplementary Material [file IMAG.a.1085_supp.pdf]

Supplementary Material for "Extracting Reproducible Components from Electroencephalographic Responses to Transcranial Magnetic Stimulation with Group Task-Related Component Analysis"

Bruno Andry Couto[1,2*],Matteo Fecchio[3,4],Simone Russo[5,6],Enrico De Martino[2], Sara Parmigiani[7], Simone Sarasso[5], Thomas Graven-Nielsen[2], Daniel Ciampi de Andrade[2], Marcello Massimini[5,8], Mario Rosanova[5], Adenauer Girardi Casali[1*]

[1]Institute of Science and Technology, Federal University of Sao Paulo, Sao Jose dos Campos, Brazil.

[2]Center for Neuroplasticity and Pain (CNAP), Department of Health Science and Technology, Faculty of Medicine, Aalborg University, Aalborg, Denmark.

[3]Center for Neurotechnology and Neurorecovery, Department of Neurology, Massachusetts General Hospital and Harvard Medical School, Boston, USA.

[4]Department of Neurology, Harvard Medical School, Boston, USA.

[5]Department of Biomedical and Clinical Sciences, University of Milan, Milan, Italy.

[6]Wallace H Coulter Department of Biomedical Engineering, Georgia Institute of Technology and Emory University, Atlanta, USA.

[7]Center for Translational Neuroscience (CNT), Department of Human Physiology, University of Oregon, Eugene, USA.

[8]IRCCS Fondazione Don Carlo Gnocchi ONLUS, Milan, Italy.

*Correspondence: brunoanc@hst.aau.dk, casali@unifesp.br

November 25, 2025

1 Supplementary Methods

In this section we describe the main steps of the Python algorithm for extracting the spatial filters w, gTRCA components y_α, and scalp maps m_α from epoched data X_α^k recorded using n EEG channels in a group of A individuals ($\alpha = 1, \ldots, A$) and K trials ($k = 1, \ldots, K$). The code is available at https://github.com/Boutoo/gTRCA.

1. gTRCA is implemented in a "gTRCA class" (function `gtrca.py`) constructed from epochs in the MNE-Python format (Gramfort, 2013) through the `gtrca.fit()` method. The first step of the procedure loads the segmented data $X_\alpha^{(k)}$, concatenates trials in the matrix X_α and normalizes the result, ensuring zero mean and unit variance for each channel across all subjects.

2. The second step of the `gtrca.fit()` method calculates the matrices S and Q and proceeds with the inversion of the Q matrix. In the case of TMS-EEG data, pre-processing steps such as average reference, bad channels interpolation and use of ICA typically result in a low-rank covariance matrix ($rank < nA$) and strategies of regularization need to be employed. The `gtrca.fit()` method applies a Singular Value Decomposition regularization in order to detect the real dimensionality of the data before proceeding with matrix inversion.

3. In the final step, the `gtrca.fit()` method derives the eigenvalues (λ) and eigenvectors (w) through eigendecomposition of the $Q^{-1}S$ matrix.

4. gTRCA components can then be extracted using the `grtrca.get_component()` method of the gTRCA class. The time-series of the components are obtained for each subject through the product of the subject's eigenvectors (spatial filters) and the epoched data. The corresponding scalp maps are calculated as the projection of the subject's covariance matrices Q_α onto the eigenvectors.

5. The `gtrca.get_component()` method returns individual components that are both normalized and oriented. Normalization is achieved by ensuring that the average time series of each subject has unit variance and zero-mean baseline. Orientation is achieved by a two-steps procedure: first, we verify that the peak of each individual component is aligned with the polarity of the corresponding peak at the group-level. Then, we recalculate the group average and reorientate individual components based on the sign of its correlation with this group average. This procedure can be applied either in the temporal domain (in which components are oriented by their time courses) or in the spatial domain (in which components are oriented by their spatial maps). By default, the `get_component()` method uses temporal orientation for averaging time courses and spatial orien-

tation for averaging spatial maps. Temporal and spatial orientation can be disabled with the options `orientation_Projections=False` and `orientation_Spatialmaps=False`, respectively.

Together with the code available on Github (https://github.com/Boutoo/gTRCA), we provide a Python function (`callgtrca.py`) that includes an example of how to fit the gTRCA model and display the results using the datasets employed in this study.

2 Supplementary Figures

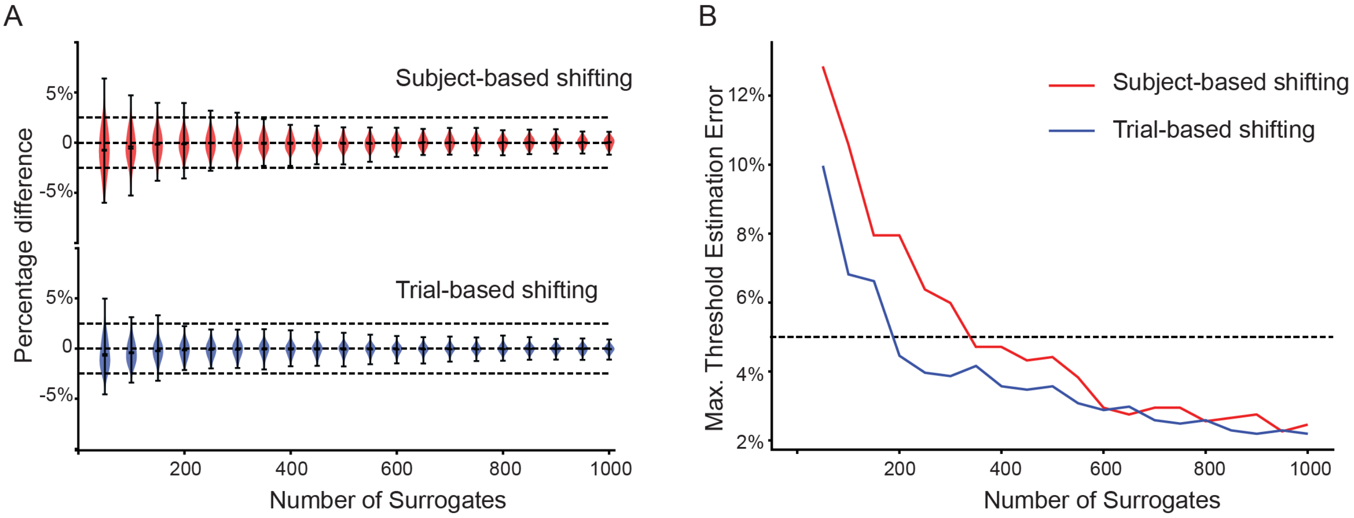

Supplementary Figure 1: (A) Error in estimating the gTRCA statistical thresholds for the Milan dataset when using a reduced number of surrogates (N). For each N, the full set of 5,000 surrogates was subsampled 200 times, and a threshold was recalculated for every subsample (subject-based test in red, trial-based test in blue). Error is expressed as the percentage difference between each subsampled threshold and the reference threshold obtained with 5,000 surrogates. Violin plots depict the distribution of percentage differences; horizontal dashed lines mark ±5% error. (B) Maximum percentage deviation observed across the 200 subsamples for each N (red = subject-based; blue = trial-based). The dashed line indicates a 5% deviation. In the subject-based test, approximately 400 surrogates ensured less than 5% error in every iteration; in the trial-based test, around 200 surrogates were sufficient. All 4,000 surrogate-based iterations reproduced exactly the same three between-subject components reported in Figure 5 of the main text.

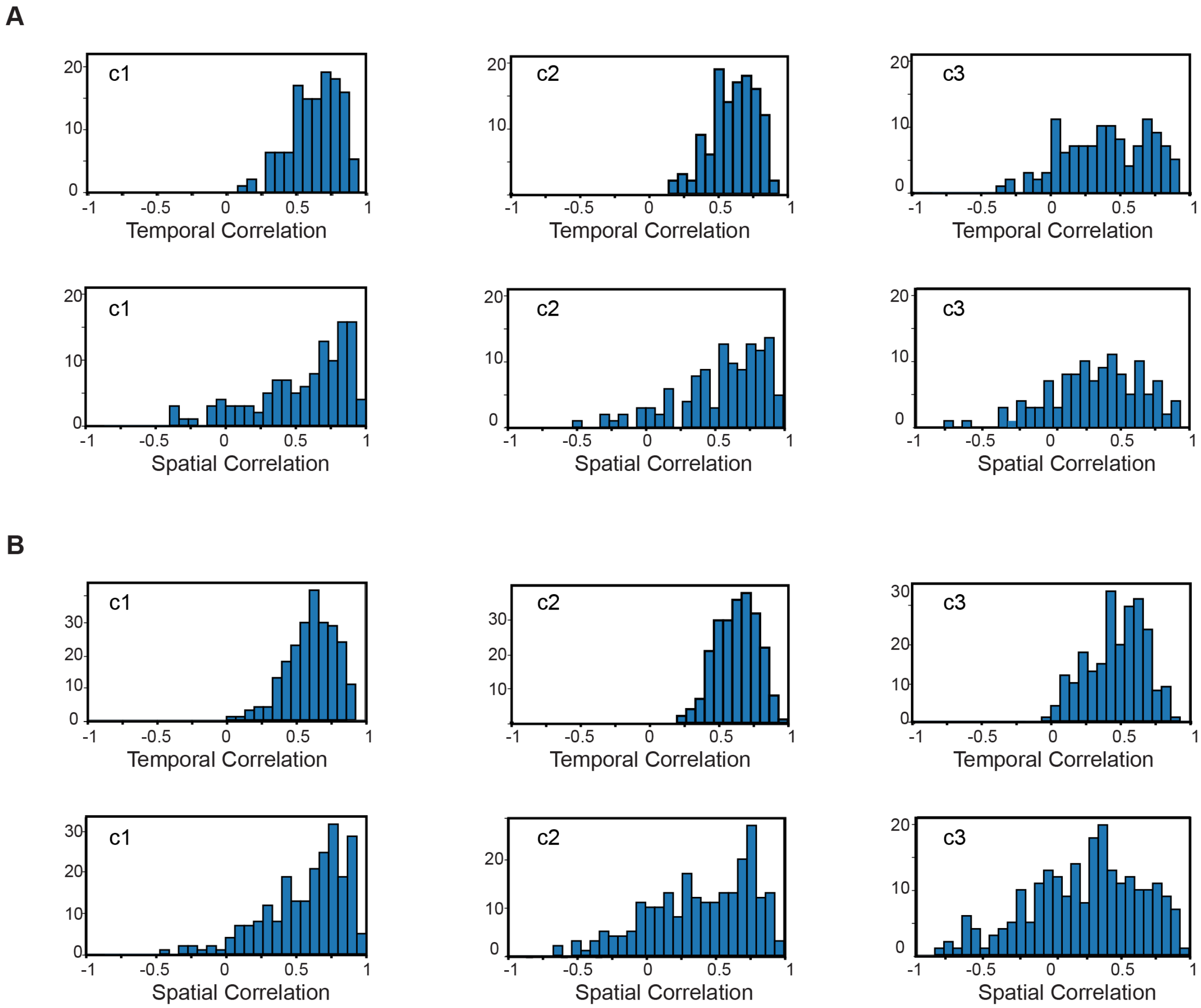

Supplementary Figure 2: Distributions of temporal and spatial correlation values for each significant gTRCA component evoked by M1 stimulation across all subjects taken pairwise both in the Milan (A) and Aalborg (B) cohorts (c1 left; c2 in the middle; c3 right). Components were oriented on time or space according to the type of correlation.

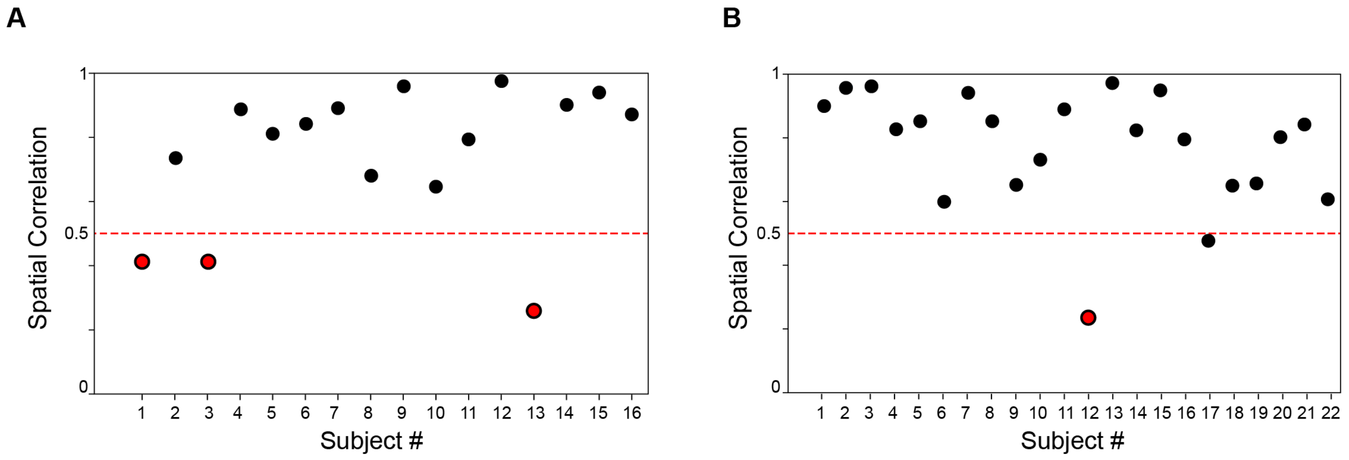

Supplementary Figure 3: Correlation between the mean topography of the first gTRCA component and the individual spatial maps for the Milan (A) and Aalborg (B) datasets. Subjects 1, 3, and 13 from the Milan cohort and subject 12 from the Aalborg cohort were identified as outliers using the IQR method (red filled circles).

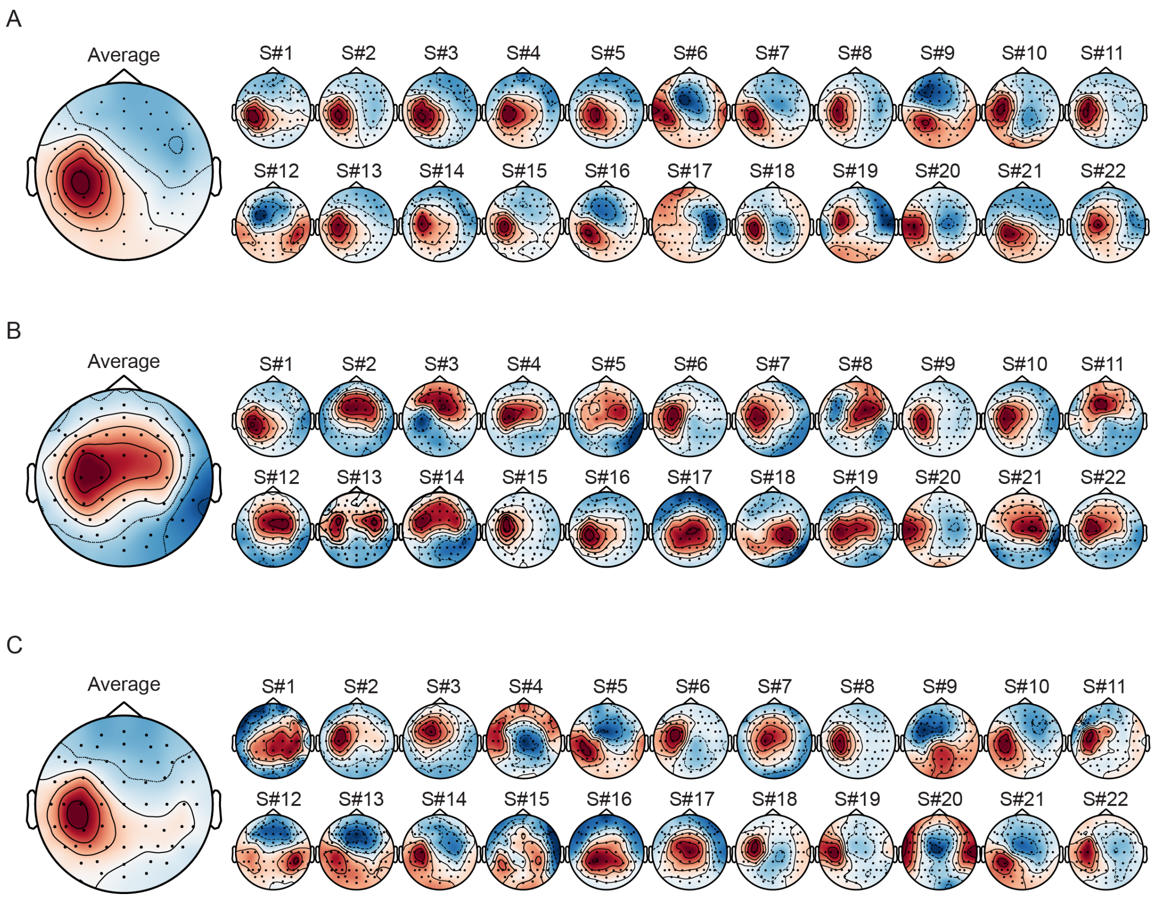

Supplementary Figure 4: Scalp maps for each significant component of the Aalborg cohort (A: c1; B: c2, C: c3). Averages across all subjects are shown in the left topographical plots. Individual topographical maps, oriented spatially, are displayed to the right (see Supplementary Figure 5B for the corresponding maps without spatial orientation).

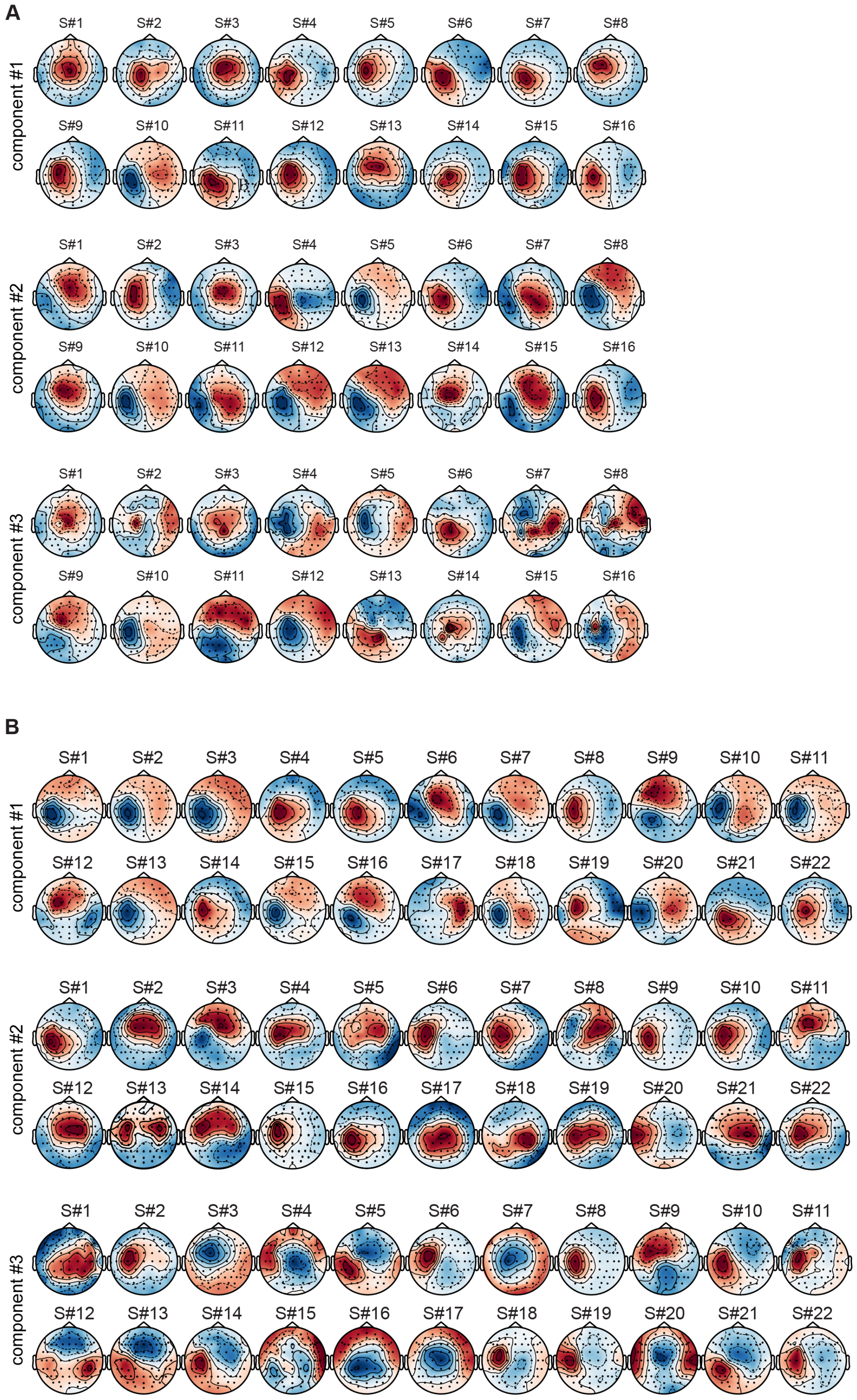

Supplementary Figure 5: Individual scalp maps for each significant component of the Milan (A) and Aalborg (B) cohorts, calculated without spatial orientation.

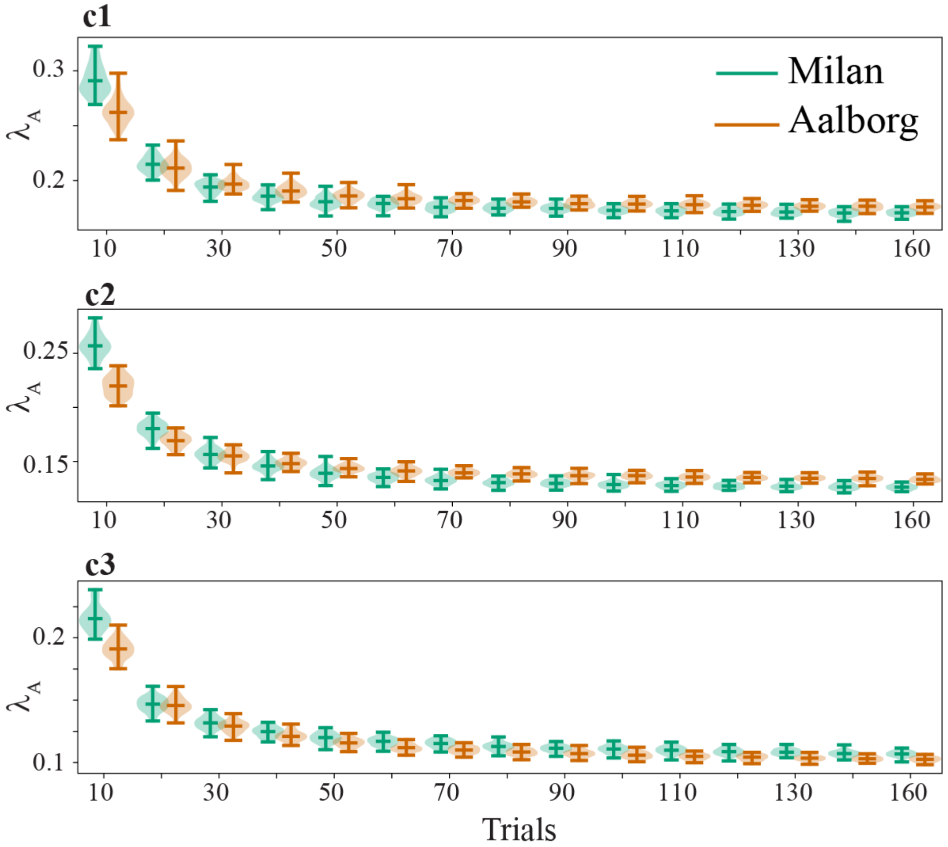

Supplementary Figure 6: Violin plots of normalized eigenvalues (λ_A) for the Milan (green) and Aalborg (orange) datasets as a function of the number of trials. Each graph shows the mean eigenvalue, along with the 2.5th and 97.5th percentiles for each significant component (c1 above, c2 in the middle, c3 at the bottom).

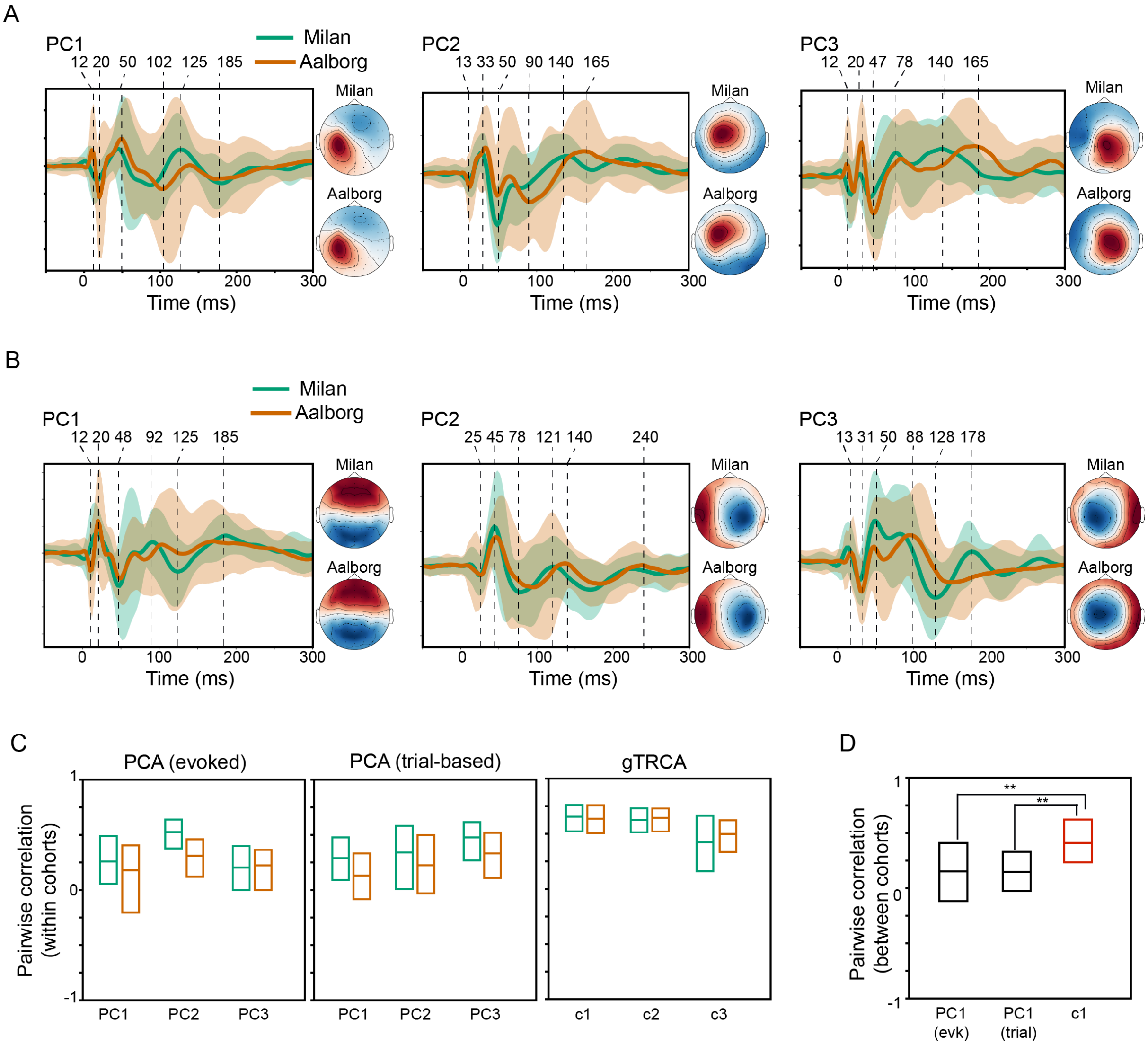

Supplementary Figure 7: Comparison of PCA and gTRCA. Principal Component Analysis (PCA) was applied to the concatenated subject-level trial averages (A) and to the same normalized trial-level data used for gTRCA (B). Panels show group-average time courses for the first three principal components (PC1: left, PC2: center, PC3: right) as solid lines, with shaded areas representing ±1.5 standard deviations for each cohort (green: Milan; orange: Aalborg). Vertical lines mark the main peak latencies. The corresponding topographic maps display the PCA transformation weights for each group. Explained variances for Milan and Aalborg in Panel A were: PC1 = 51.99% and 52.64%; PC2 = 30.43% and 30.04%; PC3 = 12.71% and 10.76%. Pearson correlations (r) between group-average components were: PC1 = 0.54; PC2 = 0.61; PC3 = 0.47. In Panel B, explained variances for Milan and Aalborg were: PC1 = 45.31% and 46.8%; PC2 = 24.3% and 22.9%; PC3 = 22.1% and 21.2%. Group-average correlations were: PC1 = 0.69; PC2 = 0.88; PC3 = 0.52. Panel C shows pairwise cross-correlations of individual components within cohorts for PCA on trial averages (left), PCA on the trial-level data (center), and gTRCA (right). Boxplots depict medians and quartiles for Milan (green) and Aalborg (orange). Panel D presents between-cohort pairwise correlations for the first principal component (black) computed from trial averages (left) and trial-level data (center), and for the first gTRCA component (red), $^{**}p < 10^{-23}$. When PCA was applied to concatenated trial averages (A), the principal components captured early M1-evoked responses with spatial patterns consistent with those described in Biabani et al., 2019. However, this trial-averaged approach implicitly assumes reproducibility across trials—a key dimension that gTRCA explicitly evaluates. When PCA was applied instead to the same trial-level data used for gTRCA (B), spatial maps and group-average components remained broadly similar across cohorts, but individual components showed much higher within-cohort variability, with less specific topographies and less interpretable temporal profiles. In both PCA approaches, because PCA maximizes explained variance rather than reproducibility, the resulting components displayed substantially lower within- and between-cohort reproducibility than gTRCA. This is reflected in the wider shaded areas in Panels A and B relative to the corresponding gTRCA components shown in Figure 8 of the main manuscript, and in significant differences between pairwise cross-correlations as quantified in Panels C and D.

Supplementary References

Biabani, M., Fornito, A., Mutanen, T. P., Morrow, J., & Rogasch, N. C. (2019). Characterizing and minimizing the contribution of sensory inputs to TMS-evoked potentials. *Brain Stimulation*, *12*(6), 1537–1552. https://doi.org/10.1016/j.brs.2019.07.009

Gramfort, A. (2013). MEG and EEG data analysis with MNE-Python. *Frontiers in Neuroscience*, *7*. https://doi.org/10.3389/fnins.2013.00267
